# Supplementary material for: NE-MTOC Formation in Skeletal Muscle Is Mbnl2-Dependent and Occurs in a Sequential and Gradual Manner
Source: Cells. 2025 Feb 7;14(4):237. doi: 10.3390/cells14040237 (PMC11853192; doi:10.3390/cells14040237)
Supplement: Supplementary file 1 [file cells-14-00237-s001.zip › cells-3412239-supplementary.pdf]

cells

MDPI

Article

NE-MTOC Formation in Skeletal Muscle Is Mbnl2-Dependent and Occurs in a Sequential and Gradual Manner

Payel Das, Robert Becker, Silvia Vergarajauregui *,† and Felix B. Engel *,†

Experimental Renal and Cardiovascular Research, Department of Nephropathology, Institute of Pathology and Department of Cardiology, Friedrich-Alexander-Universität Erlangen-Nürnberg (FAU), 91054 Erlangen, Germany; payel.das101192@gmail.com (P.D.)

* Correspondence: silvia.vergarajauregui@uk-erlangen.de (S.V.); felix.engel@uk-erlangen.de (F.B.E.)

† These authors contributed equally to this work.

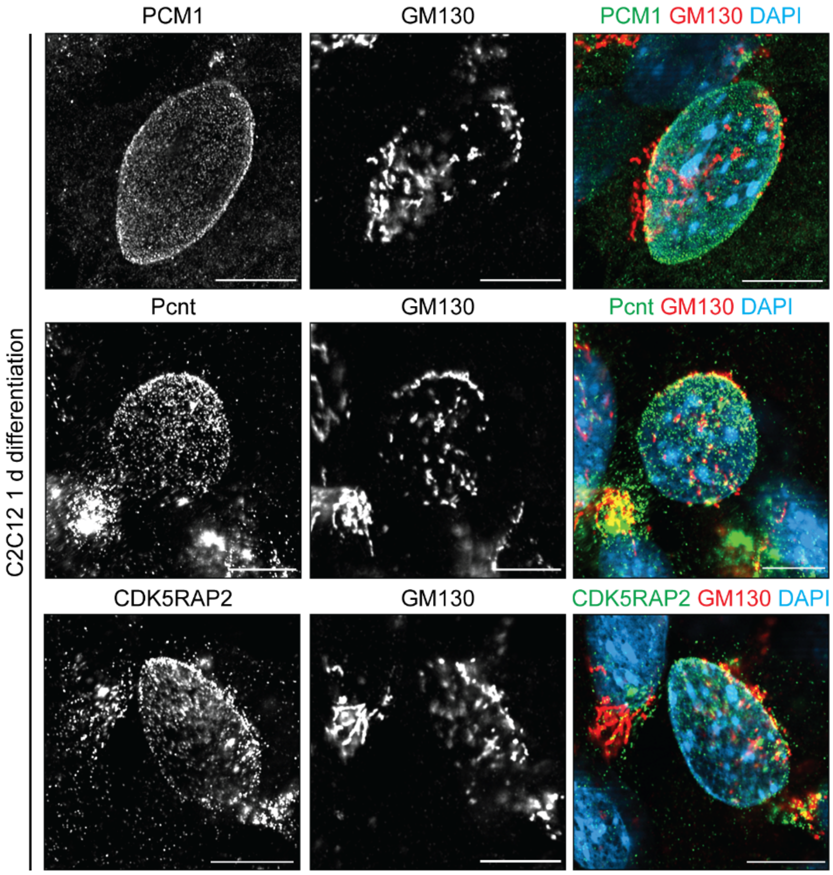

Figure S1. Golgi and MTOC proteins do not overlap. Immunofluorescence analysis of C2C12 differentiated for 1 day (d) showing co-staining of GM130 (red) with MTOC proteins (green) PCM1, Pcnt, or CDK5RAP2. Scale bar: 10 μm.

https://doi.org/10.3390/cells14040237

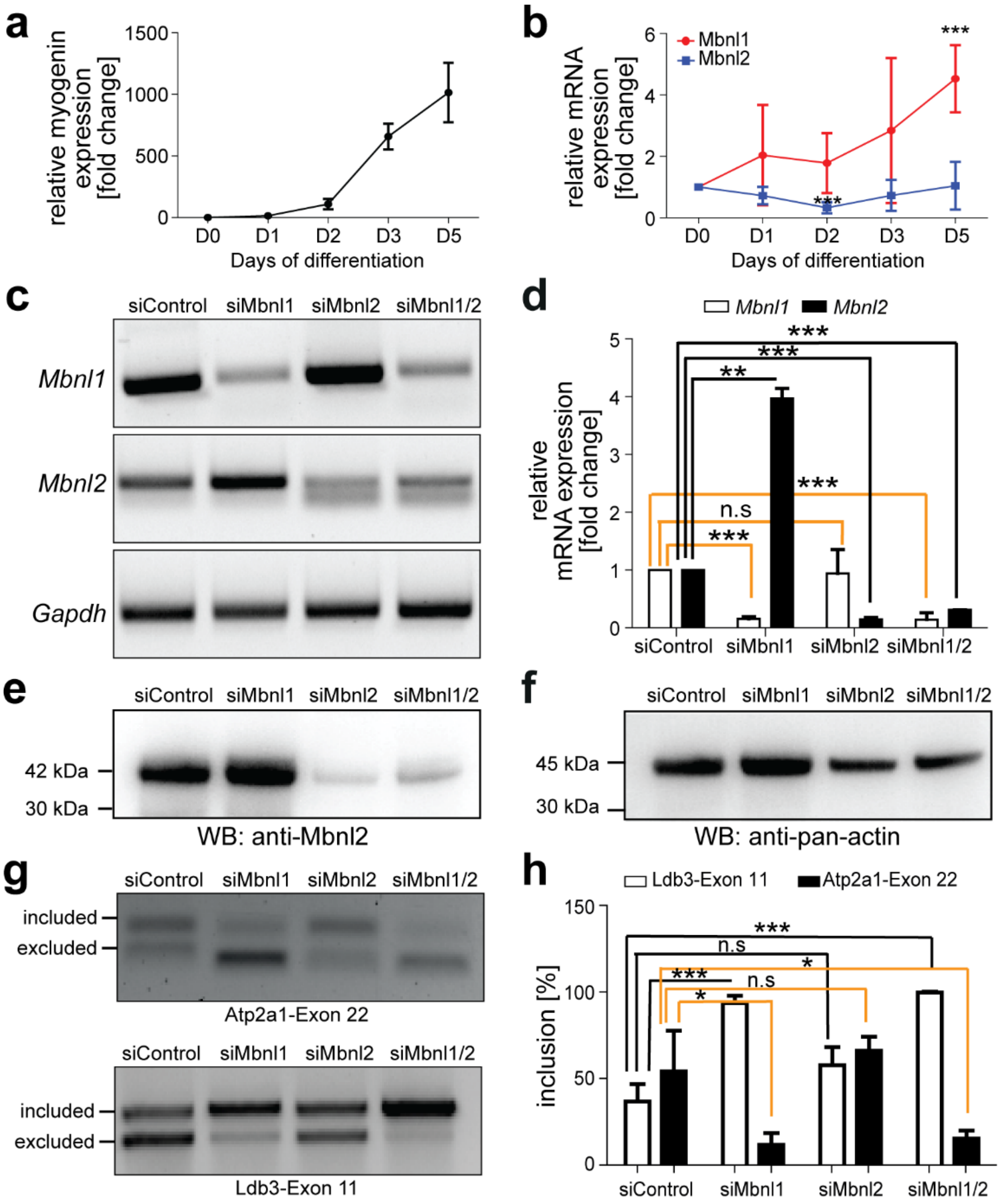

Figure S2. Mbnl1 and Mbnl2 can be knocked down by their respective siRNAs. (**a**) qPCR analysis of myogenin expression in C2C12 cells over a period of 5 days from myoblast stage (D0) to differentiated myotubes (D5). (**b**) qPCR analysis of *Mbnl1* and *Mbnl2* in C2C12 cells from D0 to D5. Data are shown as individual biological replicates with mean ± SD. $n = 3$. ***: $p < 0.001$ compared to D0. (**c**) Agarose gel of RT-PCR products visualizing *Mbnl1* and *Mbnl2* RNA expression in siControl- and siMbnl-treated C2C12 cells. *Gapdh* was used as loading control. (**d**) qPCR analysis of *Mbnl1* and *Mbnl2* expression in siControl- and siMbnl- treated C2C12 cells. Data are shown as individual biological replicates with mean ± SD. $n = 3$. n.s.: not significant ($p > 0.05$), **: $p < 0.01$ ***: $p < 0.001$. (**e, f**) Western blot (WB) for Mbnl2 and pan-actin (stripped and re-probed) in siControl, siMbnl1-, and siMbnl2-treated C2C12 cells. (**g**) Agarose gel showing splicing alteration in exon 22 of *Atp2a1* and exon 11 of *Ldb3*. (**h**) Quantification of % inclusion of the respective exons for *Atp2a1* and *Ldb3*. Data are shown as individual biological replicates with mean ± SD. $n = 3$. n.s.: not significant ($p > 0.05$), *: $p < 0.05$ ***: $p < 0.001$. b, d, h: One-way ANOVA/Bonferroni.

a Human AKAP6 3′UTR (ENST00000280979.9)

aatgtacccccctccccaagcatgaaaatcatctcactgaaaggtacgtatagtcctcatgccgtatatgtatttaaaatattgagttcaggtcagcaaaaccatggcaggagaa
aaggctagtcagaatgaccagttacggcagacagttatctgaataagacatttaggtagaagaaatgctctcaacaatgaaagtaactgtgaagcaaaagaaaaaaaaaac
agggaaaattg**acattc**ttagtgacattatctttattacataattctggcaagaatacaaaatttgaggaagtagttgaacaatttaaggcctcaaattcttccattgagggagaa
taaaaagcttatccaaatcgcaattatactccatgatcaaaatgcatgcatagtaacaatgctctccacttcagtttcctgcacagagagctgtccgttctcttcagtgcctgcaga
tgaacaatgattaggctaaaggccacatctcctgatttggcccttccttttatgcttctgtctaatgtaactatcgtcacatttgcttccccagtggaagactgtgctgtcacaactcc
taatcacggccatgtctaaggattcaaacaggaggggaaagttgatgttaaagaagaggctcttatttatccacatacagattgagaacaggctcagaaagcaaatgcaga
gagaatacatgacaagttaatggcgttttgataaaaacagaatttttgaattttgcctgaaggtgacaggctgatttccatcaaatttttaagaaagtcattccactcttcacattt
aataagacagctattgatcgataattccaagatgatgatatgtctctaaaagtgacacaaagattctgttatttcccatttctaagccccagtgatgaatatgctttctcaatcaagt
ttaaagaaaatttgagctttcaaatgtgagagtcatatttcacccaagtatgagatgcctaatgccacctccacccccccactccagaagcttaatgccgtcacctaaactcgtgct
ataggaaatgaattacagaagcaatctaccctaatttgttaacttattcaaataacagttatatacttaactatgctaaagattatctttttgtggcagattgtttatttttgatgag
cctaattactgattttctaaaagtaatttacatttattacaaatacatcttttaaaatgtgacagataaatgttgttcccataatgtattatgaaaaaagttactatttaaaaacacatta
tttaatttgaaagatgaatggcaaacatcattatttttttttcctgcaacacctgtgccctaagcttattaaaatgtgtgattctgtgtagagctgctagaaatggggactaagggg
gggcatctataccatatataccctaaaaataaactcagtatgcaaaattgaagaaaggcatcccacttactaatatagctcaactaattttctgtgtttcagactctatttacaattg
gctgcaagtgagaacaaaagtaggagagagagaaatgtattttctgtgaaacatgtccaccctataatc**acattc**tccatcagtatgcagtaaaactgaccattaagggagag
taactgcaattccataagctgcttcaaaacaagtcctattagaaccgattgtactgctggctctgccaacctccgcagaagggctggtagagcctggctgattttgttttctgaaa
ggggataaaggcttgaaagcaacaccactctgagcatgacatcatgtgactgtgaccctcaagtcaccagtcatgctgagatctggagctccagaagctctggcactggaga
agcctttgcgaagcactctccctttttttgatcctctgggaatttcttctctctcttacatagacgtagaattgaaatgaaatgtaaatgatgagcagctccatctttagacttggagg
aagtgaatgtgctggtggatctgtgtaaccctcacattttacaagcagggacaatgtcaaactagcagccagcccgtctgccagtcagcagtatttttttgttaagccccggtgat
gcctggccttatctcgggcaaagggagaaacagccagatttacttctgcagaactcacatcgcccccaagtgacttgttataggcctagagccatttaaatagccatatttact
ggtcctattcaggatttttttcagtatcatgtggacaaaacatctggatgcctgaaatttttaccagacgttagcattggataactgggtataaatagtcacatattgttaaaatact
atcaaccaggacaaattatttaatctcaaaatgttgacatgccttcagtatttaatgtcaagaatacatagtaaaaattgtttgataaatatgaggtttcatgtctacaagagcttaa
ttgttgtcattgttcttactacaagttcagaaaaagaaaaagaaaaagctgtgctttttaaagacatttgaaaaccatcagtggtgatgctctttttatcaggacagtaaaattcac
caaattccatttgcttggtttttttttttttccagaactaagttttcagatgatgaaatgattataatgccacccagaagagatataacttagaatagcagatagagtctgcaacagt
aacaaaaatgttttccagattttcaaatgtttttatttactgtgttctaattaaaaacccactgaaactgcttgagaagttttaacatgatctaacagtatgcatttgcactgatttcca
ggcatactgctgtgcttttcactattttcatttttgtgtcatgtttctataaaaatagaggggtcagttacagcaaatcacatcaaaggaaaggtaactggatagatccagatctttc
tgcctaatttggaggaagcacataatgtatgtctggagtgcctcctaggacctgcctcagagatgtactttgcgctaatctttttgctgtattctgacagttatttcttcataatcctc
atcataagcctctgatctttgatgaatggtttaaccctaaacttcaggctggataaatcccgtggtataaaaatttaactcatcatatgcggccagtgtgtgcttttcttttcctttttg
gtctgctctagtgatttggggttttgttttactatgtgtatgtgtatgagtgtggtgttttgggggggtgggttgaggagctgtatttacttacagtgcatatatttcctttagaactttt
tagctttattgccttttctactttattggtctgtaaatgtgatagttggtggccagtagattttagattttaggcaattacttgtatttgtgcatgctcaagtacgcacataaagagtttt
aaatgctgctgctcagatttacaaactgagaaagctgtaagagcaaacatttctcatgcacacagcacaattcccattctgttaattggattgacattaggtgcttaatattttcca
aagctggtcaactaggtttaagaaactacctttggtgattgtgtatgaattttgcactagacaatacaggtataatcttatcaaacttacctttaaaatcactttaataaaccaatgtt
ttgctggatgtgaagtgtctttaaattatctggcacttctgaataactagccctgctatgctaaatgacaaggatgttactgggcatcacctatcatctatctctctctctctctctct
ctcacacacacacacacacacacacacacacacacacacacacactctc**ACACCC**cagatctgtgcctcagccaattggcctgcccatttccaagtgggccaagttgaaa
gaattgattacctagagagaatttaaatgccagcatttagtaagaccagatttgcctcacctagggaatccctattctgtggccagatttcaaatgagtcatgtcctcagcatttg
aacaatgcactgggctttcttgtgttactgcattattttactataccaaatttcatcccatctgtttctgtctgtctcccccagttctttatctaatttttcttctttctccaactccccatggct
ccagccaaaccacttttttgttaaggtttttctggctcttaagttgaaagattgtagaaaaacagagagcaagacaaagagcatattcgttcttgaaggacagtaaaacaacctg
atatgagaaatcagaactcagtttaagcccagacaagcaaaagcctgctttctttgagagctggtcagggggacttacaatagttgcatcctgcattgcctaaacttggtattta
ggtgttcctcacatttgaccagttgcttttagccatttagaaacaggcagaatggatcaagtttggaagaaatttatatcatagccatgagatgtacaaatataattgaatccatct
cgtttccacaatgctgtatgtaccttaaatgaaatgcattactatttgagctagaagccccagttgtatctttttataggctgaaacacttaggataatcacaatgttgtgaatatttc
accactatacaatagtggaattgttcgatatcatgcataaaatcactgcaggaagggttaagcctgagagcctatcagctcccagtctgtggaatcattgtacattgtaaatattt
tgccgcatcacttggcatcagtggcctcacattttcatattggtttgaagctattttgatgttccaaactgagtgcacaggcaaaagagtggatgacatccagagaaaaggtca
aacttgatgatttttcttgttttctttgaaaccacgtctttttaaaaaaaaaaaaaaaaatcaatgtatttttcacatctacgctgagactttttttgtcagtaatgtgaagcttgggctatta
aaattatttatattttatgtatctttatatttcagaacattagcaagtagtcccacaattgcagccttcaatggttcctcaaagggcttggttttgctaatgaaaaatacatttctgaaa
ccttttcttgtcactttttttgtttcttgtagatacgcctggctgcaactcaggggtggcctcatcctcccgccctgggctggcctctggttccatcacgtttgtcactgccgtttattaca
ttgacttctcccaagatgaatcttccttccaaatgtgttttctccacacaagccttgtgatctgaatgtgtgcgctggttctctttaggtgatcgtctttgaagttcagcaaagctgctt
gttctcccatggattcctgtcccaagctacctctaccaaccctctctctccagctagacttttctctttgcctcctcccttcccttccactctttaaagttctgcagttcaccaactggtag
tccattaaattctcctgtctagaatgacccccccaccagtacttgaccaatttcatgtatcaatctggattttttttttaacggtataatgactgtgtttattgaaagagttttacctaaaa
agccaacatttgaattggttgcagcatagagaagaaacactggtccttctttcaaaattaagcaactattaaaagcgccattttatttatttcatttaaaaaataatctatgcagcat
ttcaagaaacaaccatatggtgttgtatattataaactggtg**acattc**tactattgaattatgtacaacattttcatttttttatgcttcttgaggtggtaatgagaaaaaagtttttttaa
aaaagtgtgccttgctgtatttcttataccatttattaaaaagctgctttcacggtaaaattatgttggtttgaaaggaggaaatagcaaggttaagatgtgtgaataatttctgtat

atatgtataaccaagtacaaacattgatgtataatgacagtataaaatgctttcatgtttgtgatgtctagtgatgtggaaaatataagccttaaatccattagattgcatggtaatt
aaaattggcataataaacacagattattgggggaaaaggaaaattagtgatctcttctactatgttctttaccaaattgttgcatctggttctgaaaaagtatagcatgtagcagc
ttccaaacatattcatattgcttaagaggcttaacattacctaaactagagactagacgtaaagccttcagttttcaaaatctttctggtcactataaagatcttggaacagcaaat
gattaaatgtcagttcccctaaaccaataaacatttatactagatttttttatttccacttatcattaatgatttaatgttggatttcaggtaccttgtatgtcttaatttattttaaatatttat
tttgaatgagtttgatagaaagctagtagaaaagtacagaaaatttgactattatttatagatttcaggtatatttatatgtgtaaaagaaattgacaaagaaatatttcatctggc
ctttactgactcctgttaaatgcagttttaaatttatatcgtaacacctacttaagtgcctgacacagtaggtattcaataaaaatttactgaattaaaggattaaattaggtgacat
ggtgacatctatccctttattttgacactaaaacatggacacaactagaaagaggtacaatgcaatataaagtcacaatagataatatatatcaaatttctaaaaggtaaagaat
gttgtgggttcatgcagtcacaggaatgacaatcattcaacagatagttcagaaacactttttatctgcaaggcactattctagatccagaagatgcaatgttgaacaaacaga
caaagccctgccctcagaaggctgtcctgcattaggaacaagtgaacacgcaaatgacatgaagtatttgttgcagagctgaggaacagagcaaatgtagtgatagaagcg
caatgagagaagcagcagtgggtacaaggaggaagaaaaagggcttgcagagagtggaaagttagtggaatattcatgaaacttcattgcaggggtaatagaagaaaa
agtaaattgggaggacttaatggaaggtcttttaaaaagttaacttggagcttctgtatgtaaaatgctaggtaataaggacactttgtacaggctgttttgcacctgattttattt
atcattagtgccacgccaagatcatttagacgatgcttatctgtaattctaccactttaataactatttgtatttttatgccccttctgatcttttccatatgtatttctaaatggataaatt
attctaggcttcttaataggtagtaatttgttcaaaagcggttttagccagacatctagttgcagtgttcaagaggattatgggggaaagagattagagataattgtctagttag
ggggcagctggagaaaataagctaagtttgcaataacagagtacacaagtatagtggcccaggatgtagtgaaagaacaaatcctagagtctttgaaatttctaagggcatt
ctagacctctgttgggatatggtattattttacatactgacacaacctaaattttctttgggtagtaactaatgtcaagtctacatcgactggtaaaacattcaaagaacaaactga
caatgatgttctacctacttgttacatgctcatggaagaccgtgcagtattgaaagtatttgttaattatctgcttagtattaacactaaatttgtagaatgactttcaggtttgttgaa
caatgccttttcaggttggaagaagaaaaatagcctcaatctcccaccccatgtaggcactacctccccaattacccttagaaaatgatcacaccaactctgcctacacacttcca
gtgatagtggctcattgtctgttaaggcaaactgttccactgttgggcatatctctttgttagaaagttctttcttaggttgctaaaatctgcctagtaccccgctaccctgttctgtct
tatggagcagcccagattatctttactccctctttctcatggcaaccctgaagataatcaaggccagttactcatcatctcccaaccactgtttcctcaactgcccttcatatgtcatg
gttttcagatccattccaacctgactgaatgttaacagacagaattcttcacattaaggaactgtcttcatcatcatacatgtagaaaagaatctgaacatttaagtgcgaagttttc
tctagaaatatattcaagatatgtttattctattattgtaaatttcaaacaataaataaataagaatccatgacttccttcagtggcccagtccagtgcctaagtcatctggaatcttct
ccttacatcgaatacaaacctaacctgtttccaagtagggcactccctccgcttatttattcatttattcagccattcagcaaacatttattaaatggctacctatgtgaggcactatat
ttggcattaggtatataaaggcaactaacacatggtatctgtctccaaaggtttacagcttttcttctgaaagtgccttttgtgtccttgaatagagtgattatgactttgtctcaatt
aaatgatgacctcatttagtttctgatgaagtttctctacatttaagaaaatctagtgagcaaaacggtatacaaaaaatcatctggatctgactaccagaaaaagtaaagtggtt
ttaattacagaaagaaaaatatttgttagatttctggtcactgaataaatatgcatcttatacaacacagaacaacctaaatttagggcttctggaaacctgagtttgatgactttg
ttgtgtttggttcatccggactaggtttcacccattctataattcctagaaacaaactagatgaaaatatcaatgaaatgatccaggtctgtcttacaagtcaatttgctttagcttcc
aaaagaagttgaaatttttagaattttaaagaaaaactcacttttcaagtgtcatataattcttatatttaaatataaatcgaaccaactaaatttaccgctatatttttttctacatat
tctttttttcaattttcattttgaaaaaattcagacctatgtaaaagttgagagtagaacaaagatacttggtactcatccctcaggttcatttattgttaatatttcaccatatttacttca
tctctgtatacacacacactttttttgtatgattaaatcatttgaaattaaattgtagagtatgatacttcatctgcaaatacttcagcatgtgttagctgagaacatgggtattcttctg
caagtctacaataccatcttcatactcaagaaatgtaacattggctggtcatggtggctcacgcctgtaatcccagcactttgggaggccaaggcgtgtggatcacctaaggtc
aggagttcgagatcagcctggccaacatggtgaaaccccgtctctactaaaaatacaaaaattagccaggcgtggtagctgtaatcccagctacttgggaggctgaggcag
gagaattgcttgaagccaggaggcagaggttgcagtgagccgagattgcaccactgcactccagcctgggcaagaagagtgaaattttgtctcaaaaaaaaaaaatgtaata
ttaatacaataatacctgatagtttatattcaagtttctctaactacccaaattatgtcctcttatagttgtttttgttttaatacagtattcaaacaaagatcacacactgcttttagtttc
caagtcttttttttttttttttttttaaatcttgcatagtttccctgtgcttttactctatttttctttgggcttttataacatcgacagttctgaaaagtccaggccagttttgcagaatgatc
cacaatcttgatgggtctagttgttttctcattgttaaactcagggtaaactttttttttgtcaagtaaatacataggtgactttgcattcacagccactctttaatgaagattgattgaa
taactgacattcctgatatgttttaaagttcacagcttactctttcagcccatgcttcatccatctaaggcaagggtcagcaaactgtggcacgtgggccaaattcagctcgttgcc
tgttctggtttgacccatttgaacaattgacgagcttcaaacaatttttacatttttttagtagttaaacaaaagaatacttcatgacatgtgaaaatcatatgaaatttaaattttggt
gtccatgtataaagttttattagaacacatccacgcacatttgtttccatagtctacggatgctttgacactatacaaagttacgtacttgcaacaaaagccatatggcctgcaaaa
cctaaaacatttactatctggatcattacagaaagttttctgatccctggtctatgatattctaaattccgacacattttcttaggatagatttccatttgcaaattcagtgtaagacat
aaacaccggcaatagttgtgttttttttttcttttacttcctagattttattgcattctcaacttgtttcatattaccttaaaaaataaagtgcacttaaagattgtttcacatccacagttt
gtttaaggatttaaatttagttcactattactgcctggctttgattgatttagagggttagtcaattacagactaaataattagttcaatcaaaaccatcagaaaaattgacacaaac
tcacttccatgcaccagtaaactggaatcctttctttaaatgtttttctaaagcaatccgaggaattaaattcaggccatttcacagtcagtttttttcaaattcatttttcaactcaaag
gcaaaaccatgatcaaaatattgtatatagtacaccttcagttcccaactgctgaatttaccaaggaattaagtattcaccacaaaccctggtgtggccataggtggacactcta
attaaaaagaagatgttcaaaaagaatagtccttcttttctttcttaaattcacctttgatagcactgccacacttctgggaagggaacgttcaaggaaaccgtatagccacttcttt
tttaaaagcagccacataccttctatataacaatggccctttagtttgagagttgccacatatttgcatttccagagtaaaataataggatgacatgggcaggtcaggcccacaat
agtatggtttctttggcaaaggcattttggccaatgccgtaagaccattctggcccttgattaatgggttctcaccaaagcagaaattacgcagagcttatttgtccttgcattttg
aagtaaatcattgcctgcataaagcaactcttttgtctcttttccattgcaacacacaggagttggaatgtgttcatacgtgtaagactgtcttagtctgcccagactgcttggactg
tgttgtttataaacaacagaatttactgctcaaagttctggagactgggaagttcaagatcaagatgctagcagattcagtgtctgaggagggctcactttctgtttcattgatgc
accttactgcatcctcatctggtggaaggggcagtgaacaagctcccttgggccccttttacgaaagcactaattccattcatgaagacttcacaacttaatcacctcccaaagg
ctccatctcttaatacgatcacattggggattaggtttcaacataccaattttgggggtacacaaatattgaggtgatagacacttccaggcttttgccaaacattagtaaacaatt
tccagtgaatactctgccccattctctcctactcaagcagagtatcttaaggcagcagattggagtgacgttactttaagggtaactttgaagccaatctaattcatactcaaaaa

agtattccaaaattttggtactctattatgggttacaaaccccttgtgacaccatatttaagagccactgctattgaggcttgcactctcttgcttgaaatgaggtgctcccaaacct
gggagatggctcccaaagaacaagagaatcaaccccatcgtgcatttatgtgctgccccaggtaatccacaggcacaaagaacttgagcttcctggtgtgttcttcagaaaaa
aaggaattcaaaaagcaaatgtaaaatggaacagaacttggcctcaaagtgatgttggaaaaatagccagtcctgatgacttggtcgaggtaacttgctttgcccactgttaa
ctacagagccatatgataatggaacatcaaggttttccagataaacccaaagccttttgtgtctataccttgtaacctgtcatgttcatcaacgttttagactgtaccattgctctttct
cccttctttattcattttatatggggacgtttgactaggatcaccagatgtagatttggtaatttttccataccattaagatgtatgataatggattttattaatttaacacaaatgcata
aaaatatgatgtaataagaaacaatcctgaaattattaaaacatttctttgtccttacttaaacctaacctgccattttaaaagacacctaactctataaacaaatgggccattacag
acgtaaaacaaatattattcagtgaaatttattcaaggacttaaaatgattgaaggcattaggaacataaaaatgcataagatcctcttttaaatcccaatattccctaaccactgg
tttataaaaaatactctaaggtttatagaattagtttggttccgttagttgatcaagttacttacactaagtcagcaattcaaaacaaataattccctctgtgtgtgtttgtttgcttgc
aaatatgtttcattttaaagtgtctttttatatagaaagccaagttggtggaatttatcttgtcattaaaatgatgtgaaaatcaaa

b Mouse Akap6 3′UTR (ENSMUST00000095737.4)

aatgtaatgcccccaagcatggaactcatctcattgaaaggtatgtatgagtatttctgctgtgtgtgtgtgtgtgtgtgtgtgtgtgtagatatgagtttacattaatagatcc
actatagaagactggtcagcatgattagctgtgacacatacacatctggataagacattgagtagaaacacagcagtggtagtcactgtgaaaggcacaaggggaccagtg
gtgacattttgacatttcaagtgacattatatttattccatcattttggcaagagcaatacaatatttgagaaaccagctgatccatttggggttttaattcctctgactagaggaga
aaatgccccgagagttgcaggcattccttgagatcaaaatgcacgttcagtagctgtattccttactgcagctcctatttagaactgccccgtgatgagcaagggtcagactga
aggctgtgcctccttccagacatgggttttcccttaggcttctgcttcatagaactagcatcccagtcactccccttaacacggcctcagaatccaaacaggagcaaaaagttga
gggaaaagaagaggctcctatttgtacacacacagatgaagcaccagcgcaaaacgcgcatattgggaggatacgtgacaagttaatggcgtctcgataaaaacagaattt
ttgaattttgcctgaaggtgacaggctgattgccatcaattttttttgagaaattcattccacttttcatatttaataagacagctattgattgataatcctgaggtggtgatacagct
ctagaagcgatacggagactctgttatctcccatttctaagacccagtggtgaatgcacttgctcaatcaagtttaaagagaaatttagctttcaaacctgagagttgtgtttcacc
aaagtatgaggtgcataacgcccctccaccccactacagaagcctaatgccgtaacctaaactcgtgctataggaaatgaattacagaagcaatctaccctaatttgttaactt
attcaaataacagttattatacttaactatgctaaagattatctttttgtggcagattgtttattttttgatgagcctaattactgattttctaaaagtaatttacatttattacaaatac
atcttttaaaatgtgacagataaatgttgttcccataatgtattatgaaaaaagttactatttaaaaacacattatttaatttgaaagatgaatggcaaacatcattatttttttcctg
caacacctgtgccctgagctcattaaaatgtgcgattctatgttgagctgctagaatgtggtgcgagggggggaatctataccatatatataccctaaaaataaactcagtatgca
aaactgaagaaaggcatcctacttactgatgtagcttcactaattttctatttcagacactatttacaattggttgcaagtgagaacaaaagagggagagagagagatgtgtttt
ctgtgagttgtggccaccctataatc**ACACCC**tccatcagtatgcagtaaaactgaccattaagggagagtaaccgggataccataagccgcctcagaacaggttctatt
agaactgattggatgcaggctctgcccgcctctgcagaaggacccagaatgcctggctgattttgttttctgaaaaaggataaaggcttgaaagcaacagcgttgtgagcatg
ccaccctgtgactgtgaccctcaagtcagcagccctgctgagatctggagcagagacgcgctggccctagtgaaggctctgggatgcattctccctgtttgatcctctgggagt
ctcttctcttcctcacacagagaaatggaattgaaatgaaatgtaaatgatgagcaattccatttttgacttgaggggaagtggtggtgctgatagatctgtgtaattgttacatt
ttacaagcatagatggtgtaaaaatatatcagccacttgcctgcccatcagaagggttctcttgtgccccacaccaggctgtactgtgggcaaagggaacaagcagccagatt
ttacttctgtagaactgacatcgcccccaagtgacttgttataagcctagagctatgtgaacagccatagattgctcctatccacctttgagggtgtgtgagcacaagtgtgagt
gcttgcgtgtgtgagtgtgtgtgtgtgtgtgcgcgcgcgcgcgtgcacatgtgcatgtacagtgtgaatgcctatgtgtacatgtgtgtttgtgcgtgagtgtatgcatgt
gtgtatgtgtgtgtatgcagtgtgaatgcatgtgtgcatgtgtgtgtgtgtctatgtctaagtaccatgttaacaaaacatgtttgtgcctgatcttttgcttagttataagcactgg
gtgtaaatttgcacggacctgtcaggtgtcgaagtgctgtcacctgagaagttattcaatctgaaaaggctgctaagctcccagaactccttgtcagggtgtgtactgaagttac
atcgagactttacaaccacatgaacttaactgtagtcagtgttccttccactctgtccaaaagcggcagaagttcttccttttaaaagccatttgggggctgtgagatggcatggc
ctttgtaccaggacatcacaattcacccaagccaatttgctggcgctcttttcccagaaatagattatcagatgaaatgactacaagtccattcgaacgtgtttacaatgtgtttag
aaccaggcagatagtctgcaaccagaacagattcttttcaaagagttctgaaaattattttatctactgtgttctaattaaaaacctctgaactcatctgagaagatttgctgtggc
ctaacagtggtgcgtttgcacgtacttccagcacactgctcgactcttcactgtatccatctgtgtgaaaatggagaggccagttagaatcacatcaaaggggaggtaactggc
tagatccagctcttcctgcctaatttggagagagcacgtggtgtaggtctggaattcctcctagggcctaagaaatgtagtttgcactaatctttttttgctgtattctgacagttattt
cttcataaccctgatcataagcctgtgatctttgatgaatggtttaaccctaaatttcaggctggataaagtctgcagtgtaaaaagttaactcctcgcatccagccagtgtgtagt
ttcgtgttttctccttgttcatctaatggtttaagggtgtgtgtgtgtgtgtgtgtgtgtgtgtgtgtgtgattacttcttttggtcttagtttcattgttttgcctacttcatcagtctatat
gagtaatggtggccacagagttttcggtttgagatgactacttgtgttgatagctgttaacacaatttagagtgcagcgcagcttttataagttgggaaaatggttggagccga
cacttctcaccacacacaacctctcattctcttactgggctgacactggggtttcccaaggctggctaagcagctctaggaaactccattgagggtttgtatattattccttctctcg
acactacacgtgtctataacatttacctttaaatcacctttgtaacccaagcttctgctggataataattgggcacttctgaataacttgctaggctatataaagattttattggataa
agtcagtctctgtctgtctgtctgtctgtctgtctgtctgtctcactctcgctctgtctcactctcactctgtctcccctccctctcttacacacacacacacacacacacacagagag
agagagagagaaagagagagagagagagagagaggatttgggcttgagcccatttggcctgacttgttcccaagtgtgccaaattgaaaggattgattacccggaggga
attcattcaaatgccagctctttactaagatgggattggcttcaccggaggattcccttgcgcagcacagcggcagattcccgatgagtcacatcctcagcatgtgagcaatgct
tccctgtgctttcttgaattactgcattattttactgtaccaaattttgttccatctgcctctatctgtctcccccatccctgttaacttcccgtatcttctcaacatcctgtttgactgtggtg
gaactacggttggaaaactttttctgacttgtgcatgaagttgtaagtttatagaaagagacaggaagacacggaagaatatttgcttctaagaggtcgagggggagggaat
accctgatagagcacttcggaatttagattaaactcaagcaggcagagacctgctttatttgagagctggtctgagaactgaaagtactttgtatcctggaatttggttgtctctc
agatttgatcagtttttgtagccatttagaaataagtaggacagaatgagcttggaagaaatttgtaccaaatcatcacacatacagagagaattgagtcagttcagtttcctgg
acctaaaatgaagcacggtgatagttgagtggtgaattctaattataccctttcctaggtaaggaccgaaagctttaggggtcatcataatgttgtgagtgttccaccgctctata

atagaacgtcccacgcagaccagtggaggaagtgtcagcctgggagccaggtatctcctggcttgtgttcgtacatcgttgcattctttggtactgactacctgatattttcttat
gggtttttttaaaagaacttgataaaattgagaccatccccagaagaatagatgaattgacgacccagagaaaagatcatgtgcttgatttttcttttttttttttgaaaacatatg
aacatactaaggcatctatataacttactgaactgaaaatgtgtttgtgtgttctgagctagtgaaagtcacatttcataacttctcatctcagatcgctagcagatgatttcccctcc
ccaggcagcttccagaaagggcttcatcctgcgagtaacagccattgctgacacctttccctgtcaccgttttctttcctgtagatagcctggctgaagctcggggctggcctga
cccaccctgggctggtcttgggttccatcctgttatcatcactgcctcctgccacgttgacttcctgaagacgaaccttccttctgaatgcggtctgtccacgtgggcctcccgatat
ggatgtgtgcattgctcctcttaggtgatcgtcttagttcctcaaagctgcttgttctcccgtggattcctgtcccaagctacctctggcaaccctctctccagcaagacttctatttc
cctctctcctccctcctccttttaaagctctgcagttcaccaaattgatggtccattaaacccactgtctggaatg**ACACCC**ctcccatcggtacttgaccaattttatgttttgct
ctgaaatttttcatgatattatgcctatgtttattgaaagagatttacctaaaaagccatcccttgatttgattgtagtatagaagagacacattgatccttatgacaaaattaagtg
atgttttgaaagtgccattttaattatttcttttaataataatctatgcagcacttcaagaaacaactataacagtgttgtataaactggt**acattc**tactattaagtttgtttttggtttc
tatgcttcttgaggtggtaatgagaaaaatgtttgttttttttttaaacggtgtgccttgctgtatttcttataccatttattaaaaagctgcttttgtggtaatattatgttggtttgaa
aggaggaaatagcaaggttaagatgcgtgaataatttctgtatatatgtataagctaatacaaacactgatgtatgacagtataaaatgctttcatgtttgtgatgtccagtgat
gtggagaatataagccttaaacccgttcgattgcatggtaattaaaattggcataataaaaatagcttattgggggaaaggaaaattaatgatctcttctacctgtgttctttacc
aaattcttgcatctggttctagaaaagaaaaaagaatcaaactccatatattagcttccaaaatatccatattgcacagaaggcttacattgcttagactacagactaggcctgaa
gccttcataattttcaaaaatctttctgtttcactataaacatcctgaaatagcaaaggtttaaaggccatttccatcaacaatgttttattctaaatgtttttatttctacttgctaatggt
ttaataatagttgtatttggagatctcttacatgacctaatttattttaaatatttgaatggttttggtggatggtatagaaaatttgattattattttatttaaactatagatttcaggtat
atttattttgttaagaaaaatcaacagaggaatattccatttggtctttttttttttgacttgaagaattcagcttttaatttatatcataactcctactaaagtgcctgacacacagta
ggtatttcatagagtttcctgaatttgagtattgagtggggttttacatatatatatttatatatgtttcctgcattaaaactagaaaaagacgtgcaaagtgaagcacatctataat
cacacatttataatcagatttccaaaagccaatagaacatgacagacatatgtagccaccgaaatgacaatcattcattcaatagattactcattagcatcttttgcctgcaagtca
ctattctatattcagggagagcagctatgaacgatgcactgcctttggaggtttatctatgtttgagacagagtgactgaccacacagacgtgtgtgccataacgtcccacagat
gaggaagaaagcaagtgtagagaaagagtcacaacgagagagacagcggtgagaggctggggcaaagagaaaggctatgggagaagaggggttagcagggcttca
ctacatgtgtgaaaagagagaaagccaaatagagagacctgcaagatgtttacagagctgacatggagcgtttgtgtgctgtgtagtaggtataggagacccagtagcttgt
gcggggctttattttatcccacaattggaactttactggcgtcctcccaggaacacttaaataatttgcgtggatttgccccttctaatctttgctgcacatatttttaaatggatgg
ctcattctaggcttttgggtatgtagtgacttcttccaacggagtcttagagagacatctagttatagtgtttatgaggatcacaaaggggagatgttagacataactatgttgta
ggggggaaagaaaggggaaattagctagatttaaggcaatgacagagtgcacagggatcgtagcctgaaatacagagaaagacagatcttagagccctgaaaagtatttg
aacatttttggacctctgttaggatatggcattattttatatactgagacaacctgaatttctcttagtaagtaatgtcaagtctaagtctattgaaaag**acattc**aaaaaattaatg
gaccatgctggactatttgcctcttttgtactcatgaagacttagaatttgaaaatatttattagttatctgcttaacactaacaccaagttgatcaggttgactcttgagtttattgat
gccttttccagccagaagagatggaagagcctcagtctcataccctgtgtagacactaacttcccatctgcatttgaaaacagctcattggtttgtattgatttgatcaacactatc
tacgcatgtcccatgacaattgtttgttatttgttaaggcaaagtatccctcttatgggcatgtgtttctgttagaatgtgctctcttaggttgctaaataaagtccacagggtacccc
tccccttttagtttggtgtattggtcctatgcctgtgctgcagagaggcccttagaagattcctggtccctctgtctcatggccgcccttcaggtaattgagaacagttactctctttc
acaggcagttcctccaactgcccttcatatatcatgacttcagatccatttcaacctgactgaatgttaaacaggcagaacccttgatgtcaagggactgtcttcatcctacatgta
gagaggtgtgtggacacttaagtgataagttttctcaaaaatatattcaagatatatgtttattctactattgtaaatttcaataaataaacaaataaacaagtgaataaaataaga
atccatgacttccttctgtggtccaggctagtgctggagtcatcttgaatcttttccttatgttggattcaaacccaacatttccatggaaaacagatctgcctttcctatcattcaccc
actaatttattcaatcattcaa**acattc**atagagtagccactagtgtgtggcatagcattgtctcacctacacaaagacaactgaagcatatgtgcttagaagtttctctctgatcat
acccttgaagataataattatgacatttcccccagattaattttccccagacccacttactttgcctcttatctatatgtctacactatcaaaaatcaggtgagcaaattgatatgtcc
tatggtctggatgaaactgtgagaagaagtgaagtggcttagtatttttcagatattttgtttgtttgtttgtttgttattcaacaaatctgcaaacaagatagcagtctgaatctgg
ggcctgtgacagcccaggtttgattatcttgttgcttggttcactcgacttggcttttcaccataatagctgagaagagagtgggtgcaaatgtcaaacagtgaggtgagccag
ctggctttcacagggagagctgtgctctcttctgaatgaaacagaaatacctgaggttccttagagtaactaacccccgagtatcagcacttgtgaagtagaacttggaccacc
aaaacacctcttttcagatacacatagcacatagctttccacattttattttggtaattttagacttatggaaaagccaagtggaacaagaacaatgtctctgcacacttcgcttag
atacatttacttttggaatttgtatctctctttctgtccatgtcacaccacatgacactcacacacacacacacacacacacacacacacacacacacacacagacatgcacata
cacgtgtgtgcacacacactaccttcttgtatgattgtaccattaaaaattcattttctgcgtacaacacttcatttataaagatgagctactatgacaaggagcaaaggcattttct
acaagtctgtaatactttacgataatacgtagtaatccttattacagtttctctacttttttttctttttacatccaaatttaatttgtatcagattctgtgacactttttctttttttttttttttttt
acagtttctctactttaagatatgctttgtgtgtgtgtgtgtgtgtgtgtgtgtgtgtgtgtgtgtgtgtgtgtgccttttaccttcaacctagtgcccaaataatgaccagataggtgtttta
gctttgtatcttcttgatctgaaacaggtttttcattctcttattccccttttggtcctcactgtattgacatttctgaagagttcagatcagttttgtagaatgagtcataacttggataa
gtcctgttatcttctcgttgtaaatctcaggatgagctggttttgtgactatagcttatgagtgatggtgtgttcagatcatggatggagtgggagtggatcacct**acattc**ttgctt
cacattttcttttgttttctcaggcagaggtcagcagatcatggcccgtgggcctgatttaacccccctgcctatttgcgtttgtcctgggaattaagaatgacgtttactttttttatgg
ggctgaaaaacaataaaaagaatgtttttgtgaaaagtgaaagtgacaagaaatgaaaatgtttgtgtcagtgactaaagccttcttggaacacaaccacacacttgtttacac
tagatttgcactagaactgcagttacttagcatagtatgtctgagatgttttcatatatgacccatgggaaactcccttctgatctctgatctatggaattataacacctgatgccatt
ctctaagaggccaatagactgcaaagtcaatgggacatagaacatgcaagcttctctttctagtttttttcacattgtcagtttcagttccatgaagagatccattg**acattc**ttttct
tttttgtttttttgtttttcgagacagggtttctctgtgtagccctggctgtcctggaactcactttgtagaccaggctggcctcaaactcggaaatccgcctgcctttgcctcccaagt
gctgggattaaaggcgtgcgccaccaccacctagctccattg**acattc**ttaagtcacagtatactgttattttctaagacagaggtcagccaacaatagccatggaccaaatag
atctttactttcgacttgtaattttagaaaaacataaagtgtactttgttttataaacaaaaagattgttctacatcattaacctaaggatttgaatttagttagttacagattggatatt

actgattgatttggaatttagtcagttacaaattggatatttagtgcaaccaagaccacaagaaaaattggtgcaccataaactcagtgtaccaataaattgagaccttttctcta
aagcagtgttaagccaggctatttcactatcatgattatttatctatttgtttttcaacccaa**acattc**acacacacacacacacacacacacacacacacacacacacacacac
acattttcatttcctggtcatatataaaaagtttcataagccaaggaaatagccatccatttgagatcctgttgtggttgtgggtagacactctcattgagtagaagagttcaatga
aattagtcctgtagttcatccactgatttgatgcaggcagctctgtcacatgtacagcaagataatggtcaagcaaaccatacagatgcttcttttaaaacagctacatgctttcca
cacagcaattacttctgcatttgagacttgccacccatttgcttgtcccaaggaaaatgatatgatatacatagatcaggtccatgataggtttcttgagcaatggcatgctgatta
ataatgcctggagaccatctgactctcccttatggactcccaccaaagcagaaagtatgcagtttgttcctccctgtgctttgaagttaatcagagtttgcataatgcaaccttcctt
cagtccttttccaggcagcatgcatgcaaaacttggatggtgtccacacatgtgagattgttttagttcggtatgactgttacgagaaaatagtttactctgggaaatttgtaaac
aagaatatttgtagcaagacaatctggaagctgagaagaccaagattggggcaacagaagattcagtgtctggtgagatcctttgctctgcttcattgatgggtgttctctgtg
cttcctcacagcgaggggtgctgaacaagcccccccccccccagccttctatgggcagttatctccttcacaacttactcaccttccaaagacttgtattagttctgttgcactggg
gactaggcttgaacatgcaaatttagagatacagacattgaaaacacagcacaggcatttgcaggcattggggcaattgtcaataagcagtttccagtgaagagtctacttgg
ctctcttcttgccacgcaagaaagggaacaacagagttgattagagtgatatggttttaaaggcagtttaaga**acattc**taatttgtttttaaagttctttttaaagttttgtatgtt
atgattgattaacaaccttgtaacatcatatttaagacacactaaataggagatgccccaaagcacagagaatgagccctatgcaaaacactgtctgagcagccccagtttatc
tacaaccgtgaggaatttgagctgtggggagtaagctcttcagaaaaaaaaaaaaaacaaacaaacaaacaaaaaaaaccccacagcattaaaagcagagatgtgaagca
ttgtgaaacttggacttcatcaccccgagaaatggccagtccaggccgtctggtctcggaaaccttgcccagcgtgaacgttggaggcagaagatgataaagatgtcccagg
tgtgcccagagtcccacacatctcaccagctaagcttctgagctcatcagctccctactgtgcagctttccttcattcatgtagcttggggaagtttgactggaagcctcaggaat
aaatttggatatttttccatatcataaggacatgcaacaatacggcttattaatttagtgcaaatgagtaatatagtctaatctaaactgtgttaaaattttaaaaactcttctttgtat
tctatctaccatttaaaacaaaacaccataaacaaatggggggtattatagatgtaaagtgttattcagcaagatttattcaaggtcttaaatgattcaggcattaagaacataaa
aatactcaaaatcctctgtaaaccccatattccctaaccgctggtttataaaaacatctggggctatagaattcgtctggttcttctagttgaccgtgttactttaaactaagtcaga
aagttgaagcaaatcattcctttcgtgtgtatttcctggcaaacgcgtgtcatttattttaaagaatctccgaataaaaagccaaattgctggaatgtaccttgtcattaaaatatgg
tgaaaagc

c Rat AKAP6 3′UTR (NM_022618.1)

agtgtaatgcccccacgcatggaaatcatctcattgaaaggtatgtatgagtactactgctgtgtgtgtgtgcatgcgcgtgtgcctttgtgtgtgaatgtgtgtgtgtgtgtgtg
cctgtgcctgtgcctgtgtgtgtgtgtgtgcctgtgcctgtgcctgtgcgtgtgtgtgtgtgtgtgtgtgtgtgtgtaagactggtcagcatgattagttgtgacacagacacatccg
gataagacatttaagtagaaacataacagtgatagccactgtgggaagcacaaggggaccagtggtggaattgtgatattatatttattccctcattttagcaagagcaacac
aatatttgagaaaatagctgatccagttgggctttaatttcttcaacgagaggagaaaaataccgtgcgagttgcaggcattccttgagatcaaagtgcatgttcagtagctac
agtccttcctgcagcgcctacttagaacttcccagggaagagtgtggatctaagtaaagactgtgtctcttcccagactgggcttttccttaggctcctgcttacgtaactagtctc
ccagtcacttcccccggggaagactgtgctgtcacagctctccatcatggcctcagaatcccaacaggagcaaaaagttgacgtaaaagaagaggcccccctagttgtacaca
cacagattgagcaccagcgcagaacgcagataccgggaggatccatgacaagttaatggcgtctcgataaaaacagaatttttgaattttgcctgaaggtgacaggctgatt
gccatcaatttttttgagaaattcattccacttttcatatttaataagacagctattggttgatcattctgagatggtgatacagcttgaacagcgacacagagattctgctatttcc
cgtttctaagccccagtgatgaatgcacttcctcaatcaagtttaaagagaaatttagctttcagatatgagagatgtattttaccaaagtatgaggtgcattacgccctccacccc
cgccacagaagcctaatgccgtaacctaaagtcgtgctataggaaatgaattacagaagcaatctaccctaatttgttaacttattcaaataacagttattatacttaactatgcta
aagattatctttttgtggcagattgtttattttttgatgagcctaattactgattttctaaaagtaatttacatttattacaaatacatcttttaaaatgtgacagataaatgttgttccc
ataatgtattatgaaaaaagttactatttaaaaacacattatttaatttgaaagatgaatggcaaacatcattatttttttcctgcaacacctgtgccctgagctcattaaaatgtgc
gattctatgtcgagctgctagaatggggtgcgagggggggaatctataccatatatataccctaaaaataaactcagtatgcaaaaccgaagaaaggcatcctacttactgatg
tagcttcactaattttctatttcagacactatttacaattgcctgcaagtgagaacaaaagggggagagagaatgtgttctctgtgagtcctgtctaccctataatc**ACACCC**
tccatcagtatgcggtaaaactgaccattaagggagagtaaccgggatcccataagccgcttcagaacaggttctattagaactgatgggatgcgggctctgcccgcctccgc
agaaggaccggaaagcctggttgattttgttttctgaaaacagataaaggcttgaaagcagcagagctgtgagcatgccaccctgtgactgtgaccctgaagtcaccagtcc
cgctgagatctggagcagagacccagcgaaggctctggaatgcattctccttgtttgatcctctgggagcctcttctcatcctcacatagagatgtggaattgaaatgcaatgta
aatgactagagattccatttttttgacttgagggaaatggaggggctgacagagctgtgtaattgtcacatcttacaagcacagacagtgtaaaaatatcagccgcttgcctgcc
catcagaagggctccgttgtgccccacaccaggctctactgtgggcaaagggaagaagcagccacattttatttctatagaactgacatcgcccccaagtgacttgttctcaac
ctagagctttgtgaacaaccatagattgctcctacctacctttgagggtatgtgagcacaagtgcatgcaagtgtgagtgcatgagtatgtgtgtgcagtatgaatgtgtgtgc
atgtctgtgtgtgtgtgtgtgtgtgtgtgtgtgtgtgtctcagtaccgtgttaataaagcatcttggtgcctgattgtttgcctagttacaagcatagggtgtaaatttgcaccagcctg
tgaagtgtcagagtactgtcacctgagaagttattcagtctgaagatgctgctgggcttcctgtctttgtcaggatgtgtcctaaggttacattgacactttacatgcatatgagct
tatttgttctcagtgttccttctacaatgtccaaaaccagcagaagttcttcctttcaaaagccatttgggggctgttagatggcattgtctttgtaccaggacaacacaatttacca
aagtcaatttgtcggagctcttttcccagaaatagactgtttacgacttggaaccatgcagatagtctacaaccagaatagattcttttcgaagacttttgaaaattatatttactat
gttctaattaaaaacctcagagcccatttgagaagattaacctaggcctaacagtgtgtgcctttgcacttctttccagcacgatgctgggcttttgactgggtccatctgcgttaa
aatggagaggtcagttatagcaaatcacatcaaagggggggcagggtgtgactggctagagccagctcttcctgcctaatttggcgggagcacatagtgtaggcctggaatg
tctcctaggacctgcctaagaaatgtagtttgcactaatctttttgctgaattctgaccgctatttcttcataatcctcatcataagcccgtgatcttagatgaacgatttaaccctaaa
tttcaggctggataaatcctgcggtgtaaaaagttaacccctcacatccagccagtgtgtagttttgtgtcttttcctaaagttcatctaatggtttaatgtgtacatggtgtgtgtgt
gtgtgtgtgtgtgtgtgtgtgtgtgtgtgagtgatttggtcttgctttcattgtcttgcctactttatcagactatacaagtgatgggccacagagttttagatttaaatgactatgtt

gatgcacgctgacacaatttaaagggcagcccagctttcataaattgagaaagtggttggagcaaacatggctcatcagtctcattcccttactggactgacactggggtttcc
gaaggctggccaagcagctttaggaaactacatgggggcttgtacattatgccttcactagacactacacttttacctttaaaccaccttactaacccactactagatgtacttct
aattatctggttctctctctctctctctctctctctctctctctcacacgtttagagagtgagacagtaggaaagaagagcctgcagaataaaatgatagacacacacacaca
cacacacacacacacacacgggggggggggagagagagagagagagagagagagagaaatttgggcttgagcccatttggcctgccttgttcccaagtgtgccaaa
ttgaaagaattggttacccggagggaattcaaatgccagctcgtttgctgagatggggttggcctcactggaggatcccctcagtgcagattcccgatgagtcacatcctcag
catctgagtaacgctgcactgtgcttccttgaattactgcattattttaccgtaccaaattttgttccatctgcctctgtctgtctcccccacccctgttaacttcctatatcttctca**acat**
tccgtttccctggggtggacctacggctggaaaactttttctgactcatacgctgagttatcatacacaaatcagtggcggaagtgttagcctgggagtgaagttcccagtctgt
gaactgtgctcatacactgtcaccccttggtactgaccacctgacattttcatacagattttaaaaaaaaaaacaacttgataaaaccgaaatcatccgcagaagatcaaatgga
ctgatgacccagagaaaagatagtcatacttgacttttcttattttttgaaaatgtatgaacatattcaagtatctatataacttactgaaccaaaaatgtgtttgtgtgttcttaga
gccttgagctagtgaaagtcacggtcatcacgtcttgtttcagatcgctagcagatgatcccgcactggcagcttctggaaagggcttcattctgcaaatgacagagacattact
gacaccttttcctgtcaccttttctttcctgtagatagcctggctgaagctcagggctagcccaatccaccctgggccggtcttgggctccatcctgttatcactgccgcctgtcac
attgactttctgaagacgaaccttccttccgaatgcagtctgtccacgtgggcctctcgacctggatgtgtgcattgcttctcttaggtgatcatcctagttccacaaagctgcttgt
tctcccgtggattcctgtcccaagctacctctggcaaccctgtctctccagcaagacttcggttttccctccccctcctcccccccttaaagctccgcggctcaccaaattgatggtcc
atcaaacccactgtctggaatgataccccctcccatcagtacttgaccaatgttatgttttgctctgaaaactttcgctgtattagaccaatgtttattgaaagagatttacctaaaaa
gcccgcccttgatttggttgcagtatagaggagacacattgatccttctaacaaaattaagtgatgtctgaaagcgccattttaattatttctttttaaataatgatctatgcagcact
tcaagaaacaactataacagtgttgtatcttataaactggt**acattc**tactattaagtttgtttttggtttctatgcttcttgaggtggtgatgagaaaaatggttttttttttaaaacg
gtgtgccttgctgtattacttatagcatttattaaaaagctgctttcatggtaagattacactggtttgaaaggaggaaatagcaaggttaagatgcgtgcataatttctgtatata
tgtataagctagtgcaaacactgatgtatgacagtataaaatgctttcatgtttgtgatgtccagtggtgtggaatataagccttaaacccgttcgattgcatggtaattaaaattg
gcataataaaaatagcttattgggggaaaggaaaattaatgatctcttctacctgtgtttaccaatttctttcatgtggttctgggaaagaaaaagaaacaaaccccatatattag
cttccaaaatatccatattgcacagaaggcttaagttgcttagactacagactgggcctgaagacttcatgattttccaaatttttctgtttcactataaacatccgaaatagcaaa
gatttctttcccctccatcaacagcattttattctgaatgtttttatttctacttgttaatggtttaaagttgtatttggagatctcttacatgccctaatttattttaaatatttgaatgggtt
tggtggatggtatagaaaatttaattattattttatttaaactacagatttcaggtgtatttattttgttaaatattccatttggtcttttggtctttttatgacttgaaagtttcagctttta
atttatatcataactcctactaaagtgcctgacacacagtaggtatttcatagagtttcctgaattagagtattgggtggtttatatatatatatatatatgagattcctgcattaaaa
ctagaaaaagatgtgcaaagtgaaccagacacagcatattatcagatttcaaaaaggaaagagaacatagccacagaaatgacaatcattcattcagtagattagcatctttt
gcctgcaagtcaccattctagattcagggagagcagctatgaccgatgcactgcctttggaggcttctgtgttagagacagagtgac

Figure S3. Zipcode sequence ACACCC mediating Mbnl2 binding is present in the 3′UTR of *AKAP6β* pre-mRNA in mice, humans, and rats. (**a-c**) 3′UTR of human (a), mouse (b), and rat (c) *AKAP6*, whereby the published zipcode sequence is marked in green and the potential zipcode sequence in blue.

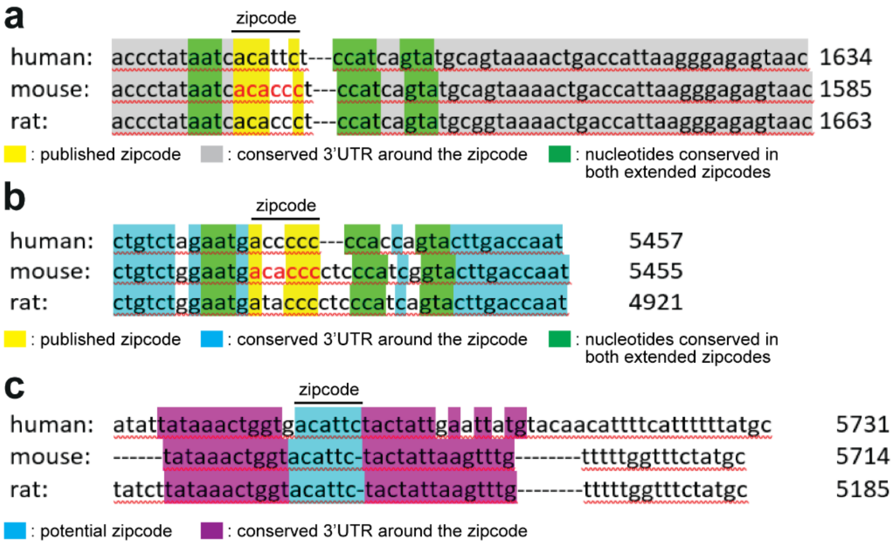

Figure S4. Sequence alignment analysis of AKAP6 3′UTRs containing a potential Mbnl2-zipcode sequence. (**a-c**) Clustal-O alignment of potential Mbnl2 binding sequences (zipcode) and the surrounding nucleotides in mouse, humans and rats.

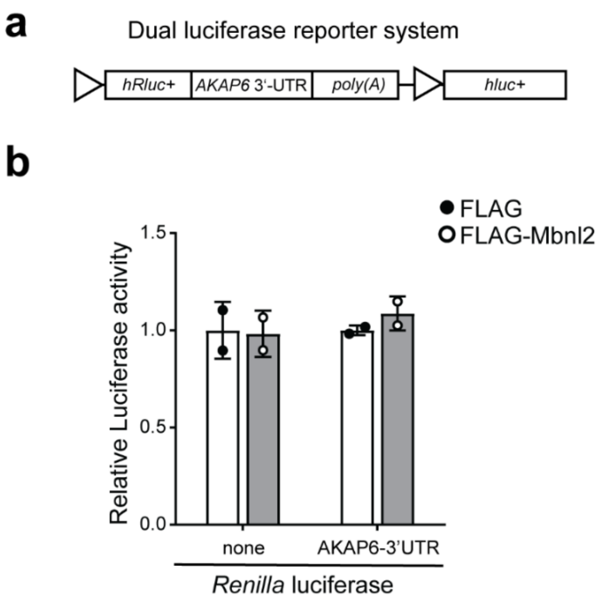

Figure S5. Mbnl2 does not stabilize the 3′UTR of AKAP6. A dual luciferase system was used to quantify the effect of Mbnl2-FLAG on the *AKAP6* 3′UTR. (**a**) Schematic of the dual luciferase system. *hluc⁺* represents the firefly luciferase coding sequence, and *hRluc⁺* represents the Renilla luciferase coding sequence. (**b**) Relative luciferase activity in HEK293T cells expressing the construct alone (none) or containing the 3′UTR of *AKAP6* in the presence of Mbnl2-FLAG or FLAG control. Relative luciferase activity was calculated by normalizing hRluc activity to hluc+ activity. Data are mean ± SD; n = 2; n.s., not statistically significant. (**c**) WB the expression of Mbnl2-FLAG by blotting with anti-FLAG antibody.
